# Physical, Barrier, Mechanical, and Biodegradability Properties of Modified Starch Films with Nut By-Products Extracts

**DOI:** 10.3390/foods9020226

**Published:** 2020-02-20

**Authors:** Marcos Leon-Bejarano, Yusuf Durmus, Maribel Ovando-Martínez, Senay Simsek

**Affiliations:** 1 Departamento de Investigaciones Científicas y Tecnológicas de la Universidad de Sonora, Blvd. Luis Donaldo Colosio s/n, entre Reforma y Sahuaripa, Edificio 7G, Col. Centro. C.P., Hermosillo 83000, Sonora, Mexico; marcos.leonbejarano@ndus.edu or leonb.marcos@gmail.com; 2 Department of Food Engineering, Faculty of Agriculture, Ordu University, 52200 Ordu, Turkey; yusuf.durmus@ndus.edu; 3 Department of Plant Sciences, North Dakota State University, PO Box 6050, Dept# 7670, Fargo, ND 58108-6050, USA

**Keywords:** pecan nut, hazelnut, nut by-products, phenolic compounds, octenyl succinate starch, starch films

## Abstract

Starch-based films with phenolic extracts could replace the use of petroleum-based plastics. In this study, octenyl succinate starch (OSS) films with pecan nutshell extract (PSE) or hazelnut skin extract (HSE) were prepared. The water resistance, as well as the optical, physical, mechanical, and biodegradable properties of these films, were investigated. The PSE and HSE improved the water resistance (decreasing the solubility to 17% and increasing the contact angle to 96.80°) and UV-light barrier properties of the films. For PSE and HSE, as their concentrations increased, the film rigidity decreased since these extracts acted as plasticizers. Micrographs obtained by scanning electron microscopy (SEM) depicted a homogeneous surface as a result of extracts dispersion through the polymeric matrix and the interactions between the phenolic compounds (PC) of the extracts and the OSS. The phenolic extracts from nut by-products and octenyl succinic anhydride (OSA) starch could be used to develop films to replace the conventional plastics.

## 1. Introduction

**Figure 1:**
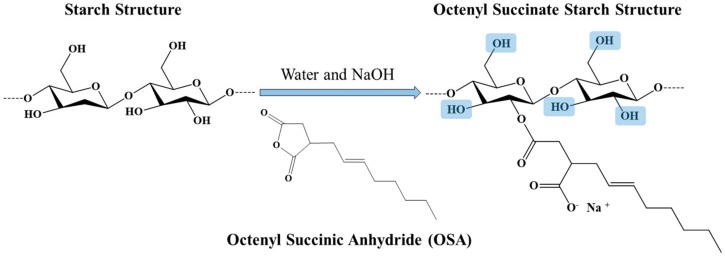
Schematic representation of the octenyl succinate starch obtention in alkaline conditions. Blue squares indicate the inclusion zone of the OSA groups. Adapted from Sweedman et al. [7].

Recently, native and modified starch has become a potential alternative for petroleum-based plastics to mitigate the environmental problems associated with these materials [1]. In this context, the food industry has been looking to use starch-based materials for the production of films that act as packaging materials or as edible coatings, thereby ensuring the quality of foods [2,3]. In addition, the biomedical industry is also interested in the implementation of starch-based materials for the development of scaffolds, wound dressings, hydrogels, and nanofibers, among others [4]. Most of the materials mentioned above are produced with native starch. However, the characteristics of native starch can be greatly improved by chemical modification, giving it greater value and expanding the field of application [5,6]. Chemical modification with octenyl succinic anhydride (OSA) (Figure 1) is one of the main modifications used in the food industry because of the emulsifying properties that octenyl succinate starch (OSS) presents [7]. 

In the specific case of film formation, the OSA group inclusion has been shown to provide greater stability, water resistance, and flexibility to the starch films [8,9]. Another highlight of the inclusion of OSA groups in the starch structure is that they improve the encapsulation capacity of bioactive compounds. They do this by providing amphiphilic characteristics to the starch [10]. It has been reported that the addition of phenolic extracts in starch-based films could undergo changes in certain properties of the films, such as optical, barrier, mechanical, antioxidant, and antimicrobial, among others. These changes can depend on the amount and nature of the compounds, as well as on their interaction with starch [11,12,13,14,15]. Nowadays, agricultural or food by-products are considered a rich source of phenolic compounds (PC) that are not commonly used [16]. In this way, the nut industry produces a large quantity of by-products (shells, skin, others) since nuts are more often consumed in their processed form [17]. For example, the shell is the main by-product generated from pecan nuts, reaching up to 50% of the total weight of the nut production [18]. In the case of hazelnut, around 2.5% of the kernel weight corresponds to the skin, a by-product obtained from the roasting and processing hazelnut products [19]. In this context, the use of these by-products could provide added value to these nuts. It may also prevent these by-products from being considered as waste and becoming a pollution problem. Both shell and skin by-products are potential sources of PC; however, the use of the phenolic extracts from pecan and hazelnut by-products in starch-based films has lacked research. Therefore, the objective of the present work was to evaluate the effect of pecan nutshell extract (PSE) and hazelnut skin extract (HSE), at a range of low concentrations, on the characteristics and properties of OSS-based films. The use of the PC-rich extracts from pecan nutshell and hazelnut skin could define an important future application for these by-products.

## 2. Materials and Methods

### 2.1. Materials

Pecan nut (*Carya illinoinensis* (Wangenh.) K. Koch) was obtained from Grupo Alta S.A de C.V., Hermosillo, Sonora, Mexico. Shells were ground in a hammer mill (Tomas Model 4 Willey-Mill) and sifted to a particle size less than 1 mm. Hazelnut (*Carylus Avella* L var. Giresun) skin was obtained at the local market from Ordu, Turkey. The skins were ground in a coffee grinder and sifted to a particle size between 0.85–0.50 mm. 

### 2.2. Preparation and Characterization of Phenolics Extracts

Phenolic extraction was carried out using the method of Liu, et al. [20], with modifications. The sample was mixed with 60% aqueous ethanol solution in a 1:30 ratio (*w/v*) and was sonicated for 15 min (Branson 1510, Bransonic, Danbury, CT, USA). The mix was centrifuged 15 min at 5000× *g* and 4 °C, and the supernatant was obtained using a vacuum filtration system. The solvent was removed using a rotavapor system (Yamato RE400, BM 200, LabConco, Kansas, MO, USA) at 45 °C. The resulting extracts were freeze-dried (Freezone 4.5, LabConco, Kansas, MO, USA) and stored at −4 °C.

Characterization of phenolic extracts consisted of the determination of total phenols and flavonoids content using the Folin–Ciocalteu [21] and aluminum chloride assay [22], respectively. For total phenols content, 30 µL aliquots of the extracts were placed in a flat-bottom 96-well microplate. Briefly, 150 µL of Folin–Ciocalteu reagent (1:10 *v/v*) and 120 µL of 7.5% Na_2_CO_3_ were added to the phenolic extract. After 30 min incubation, absorbance was measured at 750 nm using a microplate reader (Multiskan Ascent, Thermo Electron Corporation, Dreieich, Germany). For flavonoid content, 250 µL of the extract, 1000 µL of deionized water, and 75 µL of 5% NaNO_2_ were mixed and kept for 5 min. Later, 75 µL of 10% AlCl_3_ was added and kept for 1 min. Then, 500 µL of 1M NaOH and 600 µL of deionized water were added and mixed to stop the reaction. From this solution, 300 µL was transferred to a flat-bottom 96-well microplate. The absorbance was measured at 492 nm with a microplate reader. Finally, a standard curve of gallic acid (total phenols) and catechin (total flavonoids) was used to express the results as gallic acid equivalents per gram of extract (EAG/g), and catechin equivalents per gram of extract (CE/g).

### 2.3. Preparation of Octenyl Succinate Starch 

Potato starch (Sigma-Aldrich, Saint Louis, MO, USA) esterification with OSA (Dixie Chemical Company, Pasadena, CA, USA) was done, following the procedure described by Ovando-Martinez, et al. [23]. The degree of substitution (DS) was determined with ^1^H nuclear magnetic resonance, using a 400 MHz Bruker Avance III HD (Billerica, MA USA), according to Whitney, et al. [24]. The DS value of the modified starch (OSS) was 0.013.

### 2.4. Preparation of Films

Films were produced by the casting method described by Zamudio-Flores, et al. [25] and Li, Ye, Lei and Zhao [9], with modifications. The film components are shown in Table 1. 

The film-forming solution was mixed, with constant stirring (1000 rpm), on a heating plate at 95 °C for 10 min. The film-forming solution was cooled down to 60 °C, and 10 mL of the extract solution was added. Both solutions were mixed for 15 min with constant stirring (1000 rpm). The final solution was cast in square plastic plates and dried at 60 °C, for 4 h, in an air convection oven. The dried films were removed from the plate, and the thickness was measured at five points using a Mitutoyo 2416F micrometer (Mitutoyo America Corporation, Birmingham, AL). The films were stored in a 50 ± 3% relative humidity (RH) chamber until their analysis.

### 2.5. Characterization of Films

#### 2.5.1. Water Resistance Properties

The moisture content was determined by measuring the weight loss of the films according to Nouri and Mohammadi Nafchi [26], with slight modifications. First, the initial weight of the films was recorded. Then, films were dried in an oven at 105 °C for 24 h, and finally, the final weight of dried films was recorded. On the other hand, the water solubility was measured in the dried films (2 × 3 cm, previously weighed), which were placed in a flask with water (80 mL) and stirred (125 rpm, 1 h) at 25 °C. The flask content was filtered with a steel mesh (45 microns). The solid material was dried at 65 °C in the oven overnight, and the final weight was recorded. The moisture content and solubility were calculated using the following equation.

(1) $$\frac{\left(\mathrm{Initial}\;\mathrm{weight}-\mathrm{Final}\;\mathrm{weight}\right)}{\mathrm{Initial}\;\mathrm{weight}}\;\times \;100$$

The contact angle of a droplet of water was measured to determine the surface hydrophobicity of films using a Dynamic Contact Angle Analyzer by First Ten Angstroms 125 (First Ten Angstroms, Portsmouth, VA, USA) with a CCD camera, according to the ASTM-D7334-08 [27]. The contact angle was determined with Ten Angstroms 32 Video 2.0 Software (First Ten Angstroms 2000, Portsmouth, VA, USA). 

To obtain the water vapor permeability of the films, the water dish method was followed [28]. Films were placed between two metal plates with inner diameters of 5.4 cm. The metal plates with the films were placed over Petri dishes containing 20 mL of water. The separation between the water and the films was 13 mm. Using parafilm, the metal plates and the Petri dishes were held and sealed. The whole assembly was weighed with a Mettler Toledo New Classic MF analytical balance (Model No. ML203E/03). Mass, temperature, and RH were recorded at 0, 1, 3, 6, 12, 24, 48, and 72 h. Water vapor permeability was calculated according to the ASTM-E96/E96M-15 [26]. 

#### 2.5.2. Optical Properties

Optical properties, including color, transparency, and UV-vis absorption, were obtained according to Kanmani and Rhim [29], with modifications. Color parameters (L*, a*, b*) were obtained using a MacBeth Color Eye 7000 Spectrophotometer with Pro Palette 5.0 software. Films were placed on a standard white calibration surface (L* = 95.69, a*= −0.90, and *b=* 1.94). The following values were measured using the CIELAB scale: L*: 0 (black) to 100 (white), −a* (greenness) to +a* (redness), and −b* (blueness) to +b* (yellowness). The color difference (*ΔE*) was obtained according to the equation below:

(2) $$\sqrt{{\left(\Delta L\right)}^{2}+{\left(\Delta a^{*}\right)}^{2\;}+{\left(\Delta b^{*}\right)}^{2}}\;,$$

where *ΔL = L* of octenyl succinate starch film –*L* of octenyl succinate starch film with the extract. Δa* = a* of octenyl succinate starch film –a* of octenyl succinate starch film with the extract. Δb* = b* of octenyl succinate starch film –b* of octenyl succinate starch film with the extract.

Transparency and UV-vis absorption of films were obtained using a Hach DR/4000 UV-vis Spectrophotometer (Hach, Loveland, CO). A rectangular piece of film (1 × 4 cm) was placed in a quartz cell, and the transmittance (T) was measured at 280 and 660 nm. Similarly, the UV-vis absorption spectrum was obtained in a wavelength range of 200 to 800 nm.

#### 2.5.3. Mechanical Properties

Puncture resistance was determined according to the ASTM-D7192-18 [30] method, using a TA-XT2i Texture Analyzer (Scarsdale, NY, USA), with a 2 mm diameter flat head stainless probe (TA-52, Texture Technologies Corp., Hamilton, MA, USA), and extensibility platform (TA-108N, Texture Technologies Corp.). The films were placed between two polycarbonate disks with an inner diameter of 15 mm. The puncture was performed at 33 mm/s speed. Values were obtained using a Texture Exponent 32 software (Texture Technologies Corporation, Hamilton, MA, USA).

Tear resistance was determined according to the ASTM-D1004-13 [31] method using an Instron 5545 tensile test machine with a 100 N load cell. Testing conditions were done at a 51 mm/min strain rate, with an initial grip separation of 25.4 mm. Results were obtained with Instron Bluehill 2.1 software (Instron, Norwood, MA, USA).

Tensile properties, tensile strength, elongation at break, and Young’s modulus were determined according to the ASTM-D882-18 [32]. The equipment, testing conditions, and results were done, as mentioned in the tear resistance determination. 

#### 2.5.4. Biodegradability 

The biodegradability test consisted of measuring the C-CO_2_ release by the soil microbiota, according to Anderson and Simsek [33], with slight modifications. The carbon (C) content was obtained using a Primacs TOC Analyzer (Model CS22, Skalar Inc, Buford, GA, USA). In glass jars, films (400 mg) were placed in the center of 100 g of soil (pH: 6.8 and 10% moisture). Soil without a film was used as a blank. On the top of the soil, a cup with 1 M NaOH (20 mL) was placed. The closed jars were stored in darkness and were motionless until analysis. On the 15th, 26th, 41st, 63rd, 81th, 99th, 120th, and 145th day, the cups of NaOH were removed and replaced by new cups with fresh NaOH. The removed NaOH solution from the flask was mixed with 25% BaCl_2_ (5 mL) and 0.1% phenolphthalein (20 μL) and then titrated with 1 M HCl to determine the CO_2_ produced. To calculate the total amount of biodegradable material in the films, the following equation was used:

(3) $$\mathrm{Released}\;C\;\mathrm{per}\;100\;g\;\mathrm{of}\;CO_{2}=\left(\frac{1}{mg\;CO_{2}}\right)-AB\times Acid\;molarity\times Eq.\;g\;C-CO_{2},$$

where A: the amount of HCl spent in reagent blank (mL), B: the amount of HCl spent in the sample (mL), C: acid molarity of HCl (M), Eq.g C-CO_2_: equivalent gram C-CO_2_.

Finally, the biodegradation percentage was calculated with the following equation:

(4) $$\frac{CO_{2}soil\;sample-CO_{2}\;soil\;blank}{mg\;of\;C\;in\;film}\times 100,$$

#### 2.5.5. Morphological and Structural Analysis 

Surface and cross-section micrographs were obtained using a JEOL JSM-6490LV scanning electron microscope (JEOL USA, Peabody, MA, USA), with an accelerating voltage of 15 kV. The surface analysis consisted of placing the films on cylindrical aluminum mounts with XYZ conductive tape (Electron Microscopy Sciences, Hatfield, NJ, USA). In the case of the cross-section analysis, films were frozen with liquid nitrogen, fractured, and placed in aluminum mounts with carbon adhesive tabs (Ted Pella Inc., Redding, CA, USA). All films were coated with gold using a Cressington 108auto sputter coater (Ted Pella Inc., Redding, CA, USA).

Surface topography was analyzed using a Veeco Dimension 3100 Atomic Force Microscopy (AFM), according to a modified version of the ASTM-E2382-04 [34] method. Films were attached in 12 mm diameter disks Ted Pella, Incorporated (Product No. 16208). The cantilever used was a Golden Silicon Probe NSG01 (reflective side: Au; tip height: 14–16 μm; tip curvature radius: 10 nm; chip size: 3.4 × 1.6 × 0.3 mm, cantilever length: 125 ± 5 μm; cantilever width: 30 ± 5 μm; cantilever thickness: 1.5–2.5 μm). 

Interactions between OSS and PSE or HSE on films were analyzed with Fourier Transform Infrared (FTIR) spectroscopy. Extracts and film spectra were obtained using an FTIR (Thermo Scientific, Nicolet 8700, Wilmington, DE, USA) between 4000 and 700 cm^–1^ wavenumber range and 4 cm^−1^ resolution with 128 scans.

#### 2.5.6. Statistical Analysis

Analysis of variance (ANOVA) with Tuckey’s test was used to determine significant differences (*p* ˂ 0.05) between films using IBM SPSS Statistics 23 software (IBM Corp, Armonk, NY). 

## 3. Results and Discussion

### 3.1. Characterization of Phenolic Extracts

Total phenols and flavonoids content were significantly different (*p* < 0.05) between phenolic extracts, being HSE, the one with the highest concentration (Table 2). In the case of phenols content in PSE, the values were higher than those reported by Kureck, et al. [35] (186.02–275.24 mg GAE/g of extract), and similar to those reported by Contini, et al. [36] (680.3 ± 11.6 mg GAE/g of extract). On the other hand, the flavonoid content corresponded to approximately 35% and 62% of the phenolic content for PSE and HSE, respectively. That agrees with the reported by de la Rosa, et al. [37] in pecan nutshells (30%–40%) and Taş and Gökmen [38] (60%) in hazelnut skins. These results indicated that both nut by-products were a potential source of PC, and the extraction method used was suitable to obtain them. Besides, the differences in the total phenols and flavonoids content could help to explain the differences observed in the properties of the OSS films after the addition of the extracts. 

### 3.2. The Thickness and Water Barrier Properties

The film´s thickness varied from 0.087 mm to 0.091 mm without presenting significant differences (*p* ˃ 0.05). Among films, OSS films with 0.100% of PSE, 0.075%, and 0.100% of HSE were significantly (*p* < 0.05) thicker than the other films (Table 3). According to Prietto, et al. [39], and Knapp, dos Santos, Pilatti-Riccio, Deon, dos Santos and Pinto [15], the thickness increases by the solid material provided for the extracts. On the other hand, all films had a moisture content of approximately 30%, without any significant (*p ˃ 0.05*) effect caused by the addition of extracts. Similar behavior has been reported for cassava starch films with rosemary extract [13]. Likewise, Feng, et al. [14] reported that the addition of tea polyphenols to hydroxypropyl starch films did not affect the moisture of the films. In this study, all of the films presented a high moisture content, which could be related to the water absorption capacity provided by the OSA groups to the starch granules [23]. 

In the case of water solubility, the OSS films with 0.075 and 0.100% of PSE, 0.050, 0.075, and 0.100% of HSE presented a lower solubility of about from 17%–18%, compared to the OSS film (20.3%) (Table 3). Similar results were reported in sweet potato OSA starch films with oregano essential oil, by Li, Ye, Lei and Zhao [9]. According to Ghasemlou, et al. [40], the hydrophobic compounds, present in the essential oils, reduce the solubility of corn starch films. Nisa, et al. [41] related the decrease of solubility with the structural strength of the films. Consequently, this is caused by the interactions between the PC present in the green tea extract and in the potato starch. Therefore, the changes in the solubility properties are carried out by the hydrophilic/hydrophobic nature of the PC present in the extracts, and to the interactions between the PC and the starch structure. 

The contact angle values for the OSS films are reported in Table 3. Values indicated that the addition of the PSE and HSE significantly (*p* < 0.05) increased this parameter. According to Vogler [42], the film surface is considered hydrophilic when it presents a contact angle <65 °, and hydrophobic when it presents a contact angle >65 °. In this study, the contact angle of the films gradually increased with the addition of the different concentrations of the extracts, and only the OSS and OSS with 0.025% of PSE films presented a hydrophilic surface. Increases in the contact angle of starch films, with the addition of yerba mate, rosemary, and propolis extracts, have been reported previously [12,13,43]. This could be related to the interaction between the hydroxyl groups of the PC and the starch, whereby the amount of free hydroxyl groups, which could interact with the water, is limited. In reaction, the hydrophobicity of the films increase [14]. For this reason, the solubility of the films with the increase of PSE and HSE concentration decreased, while their hydrophobicity increased. 

Finally, the addition of both extracts did not cause significant changes (*p ˃* 0.05) in the water vapor permeability of OSS films, presenting values of 2.46–2.62 (g mm/h m^2^ KPa) (Table 3). Similar behavior was reported in thermoplastic starch films with a low concentration of terpenes [44], cassava starch films with propolis extracts [11], and rosemary extract [13]. These authors mentioned that the water vapor permeability could be influenced by factors, such as the starch botanical source, the hydrophilic/hydrophobic nature of the extract components, defects in the film’s surface, and others. Then, the lack of change in this property could be related to the decrease of the solubility and the increase of the contact angle due to the interactions between the PC of the extracts with the structural network of the OSS.

### 3.3. Optical Properties 

The OSS film was colorless, whereas the addition of both extracts imparted color to the OSS films, as shown in Table 4. The a* and b* values increased with the addition of both extracts, but the OSS films with HSE presented more significant differences (*p* < 0.05) than the OSS films with PSE. This could be related to the highest concentration of phenols and flavonoids present in this sample (Table 2). Contrary to a* and b* values, the L* values decreased with the addition of PSE and HSE. On the other hand, the highest color difference (*ΔE*) was observed in the OSS film with 0.100% of HSE, whereas the lowest values were observed in the OSS film with 0.025% of PSE.

Similar behavior was reported in corn starch film with essential oils [40], cassava starch film with yerba mate extract [12], and adzuki bean starch film with cocoa nibs extract [45]. These results indicated that the type and concentration of the extract has an important role in the color of films.

The transparency of the films was measured in the UV and visible regions at 260 nm and 660 nm, respectively (Table 5). Film thickness is an important factor in these values. However, due to the lack of differences between the thickness of the films (Table 3), this is not considered a determining factor in these values. According to the method used, high transmittance indicates high transparency. As can be observed, the OSS film was transparent, and the addition of PSE and HSE decreased transparency. At higher extract concentrations, a decrease in the transparency of the film was observed. A decrease of the transmittance values at the visible region (T_600 nm_) of thermoplastic starch films with high concentrations of terpenes was reported [44]. In addition, Knapp, dos Santos, Pilatti-Riccio, Deon, dos Santos and Pinto [15] and Qin, et al. [46] observed a decrease in the transparency of cassava starch films containing yerba mate extract and *Lycium ruthenicum* Murr anthocyanins. Like what was seen for color properties, the HSE had a greater impact on the transparency in the UV region (T_280 nm_) than the PSE. Probably, this could be a consequence of the highest concentration of PC detected in HSE (Table 2). So, it indicated that a higher PC content resulted in a higher UV-light absorption or lower percentage transmittance. 

**Figure 2:**
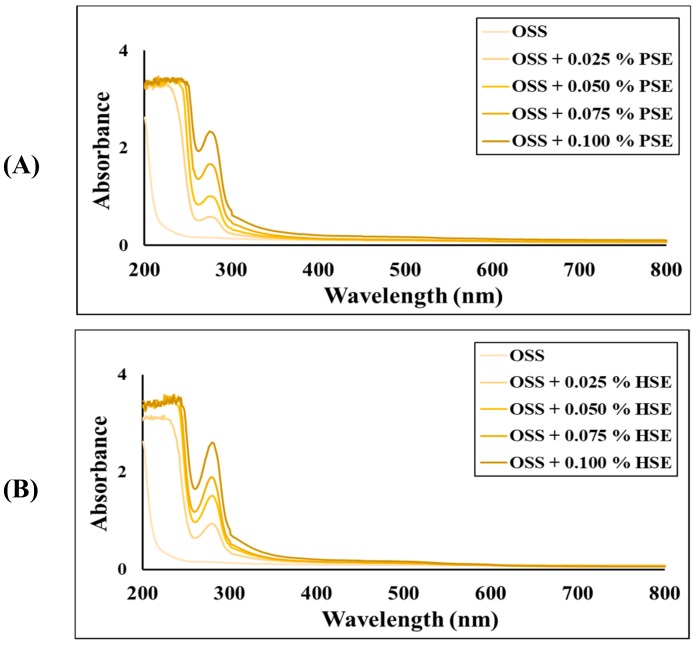
UV-vis absorption spectra of octenyl succinate starch films with pecan nutshell extract () (**A**), and octenyl succinate starch films with hazelnut skin extract () (**B**). OSS: Octenyl succinate starch, PSE: pecan nutshell extract, HSE: hazelnut skin extract.

According to these results, the addition of HSE or PSE to the OSS films improved their UV light barrier property. This could be demonstrated by the absorption spectra depicted in Figure 2. The control film did not present absorption in the wavelength range of 200–800 nm, indicating that light completely passed through the film. However, the OSS films with HSE and PSE showed absorption peaks between the range of 250–300 nm, and the intensity of the peaks increased with increased extract concentration. Some authors reported similar results and concluded that PC and pigments present in different extracts are responsible for the improvement of the UV-barrier properties of films [13,15,29].

### 3.4. Mechanical Properties 

The addition of PSE and HSE affected the mechanical properties of the films (Table 6). Puncture and tear resistance significantly (*p* ˃ 0.05) decreased with the addition of PSE. This indicated that PC from PSE acts as a plasticizing agent in the structure of the film, and decreases the film rigidity [11,47]. The rigidity of these films was lower than that reported in cassava starch films with propolis extract [11]. 

On the other hand, tensile strength decreased with the addition of both extracts, but there were no significant differences (*p* > 0.05). The same trend has been reported in cassava starch films with propolis extracts [11], and corn starch films with red cabbage anthocyanins [39]. In this study, tensile strength values were higher than what has been reported for other films [9,39,43], indicating these films are more resistant to tearing. The elongation at break is a parameter that indicates the plastic behavior of the material. All films showed an elongation at break around 30% without significant differences (*p* > 0.05). The OSS films without and with PSE or HSE had more plastic behavior than other films [11,45]. It was observed that, except in the case of OSS films with 0.025 and 0.050% of HSE, the addition of extracts significantly (*p* < 0.05) decreased Young´s modulus. High values of Young´s modulus indicates the rigidity of the films. In this case, a decrease in Young´s modulus indicated that the plasticizing effect of HSE and PSE was due to the structural discontinuities in the polymer matrix, which is caused by interactions between PC and starch [11].

### 3.5. Biodegradability 

**Figure 3:**
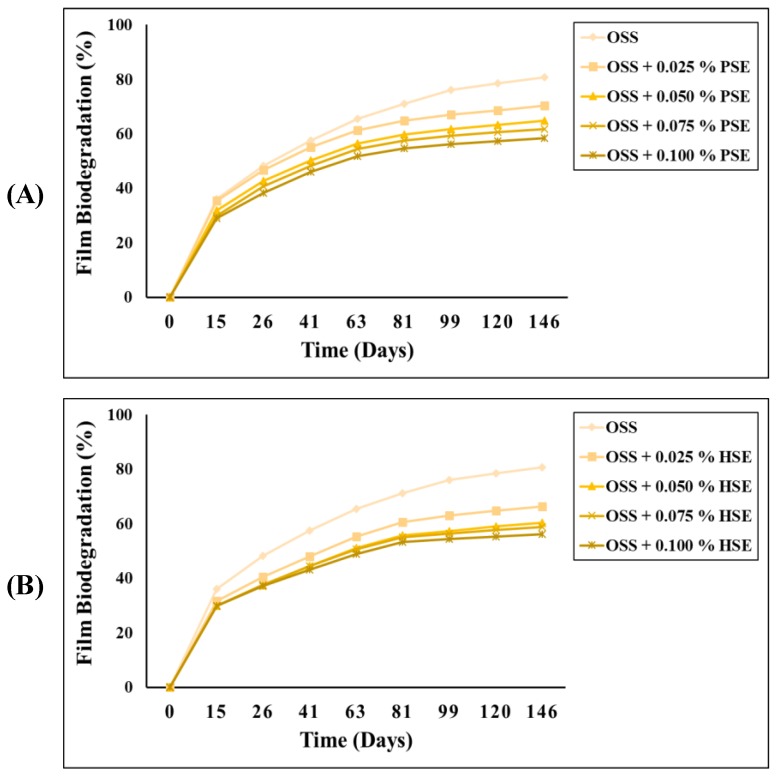
Biodegradability of octenyl succinate starch films with pecan nutshell extract (**A**) and octenyl succinate starch films with hazelnut skin extract (**B**) films. OSS: Octenyl succinate starch, PSE: pecan nutshell extract, HSE: hazelnut skin extract.

The biodegradability of the OSS films with PSE and HSE in the soil is depicted in Figure 3. The OSS films showed a higher percentage of film biodegradability compared to the OSS films with PSE or HSE. After 146 days in soil, the biodegradation value of the OSS film was around 81%, while the values for the OSS films with PSE or HSE were decreasing with the increase of the extract concentration. The biodegradation values did not exceed 71%. These results were opposite to those reported by Medina Jaramillo, et al. [48] and Kim, Baek, Go and Song [45], in which the extracts accelerated the degradation of starch films. According to these authors, the moisture and soil microbiota activity and its composition play an important role during the degradation process of films. However, they associated such an increase in the biodegradability values to the low molecular weight of some PC, which makes them more susceptible to soil microbiota. Another parameter involved in the biodegradation of films is related to their solubility. The greater the solubility, the higher the degradation by the soil microbiota. According to this, the decrease in the biodegradability of OSS films with PSE or HSE could be due to their low solubility (Table 3). On the other hand, the PC in both extracts could help to increase the integrity of the OSS films. The PC could do this by providing greater structural stability to the starch, but also increases the resistance of films to the degradation by soil microbiota [13]. Although biodegradability values were slightly higher in OSS films with PSE or HSE, they represented an option to the replacement of petroleum-based plastics, as they are much more readily biodegradable than conventional plastics. 

### 3.6. Morphology Analysis 

**Figure 4:**
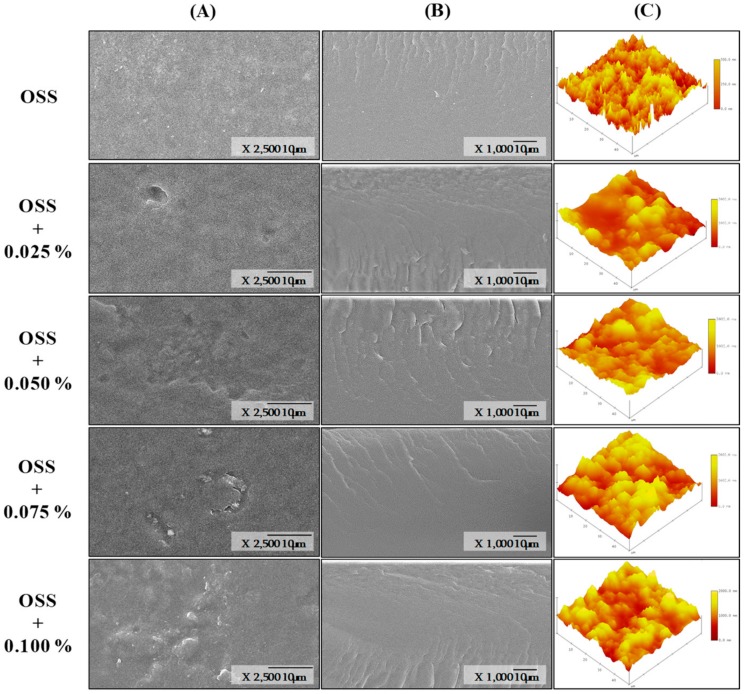
Scanning electron micrographs of octenyl succinate starch films with pecan nutshell extract, surface (**A**), cross-section) (**B**), and surface topography (**C**). OSS: Octenyl succinate starch.

**Figure 5:**
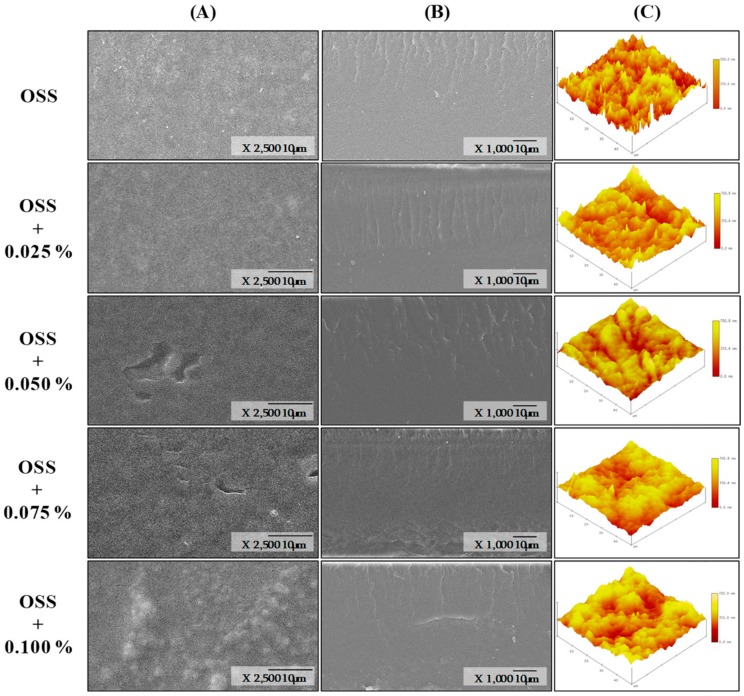
Scanning electron micrographs of octenyl succinate starch films with hazelnut skin extract, surface (**A**), cross-section (**B**), and surface topography (**C**) OSS: Octenyl succinate starch.

**Figure 6:**
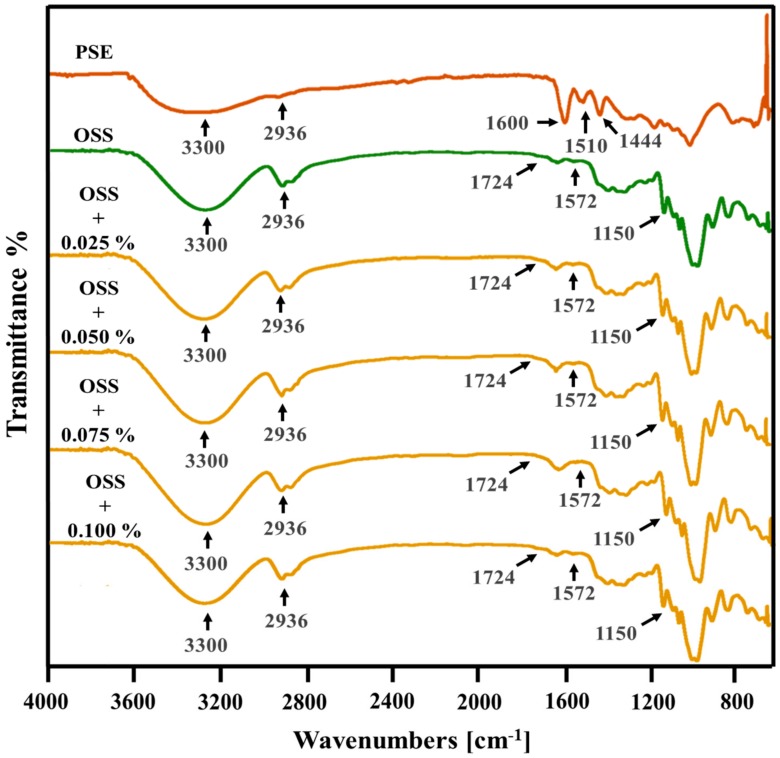
Fourier transform infrared (FTIR) spectra of pecan nutshell extract and octenyl succinate starch films with pecan nutshell extract. PSE: Pecan nutshell extract, OSS: octenyl succinate starch.

**Figure 7:**
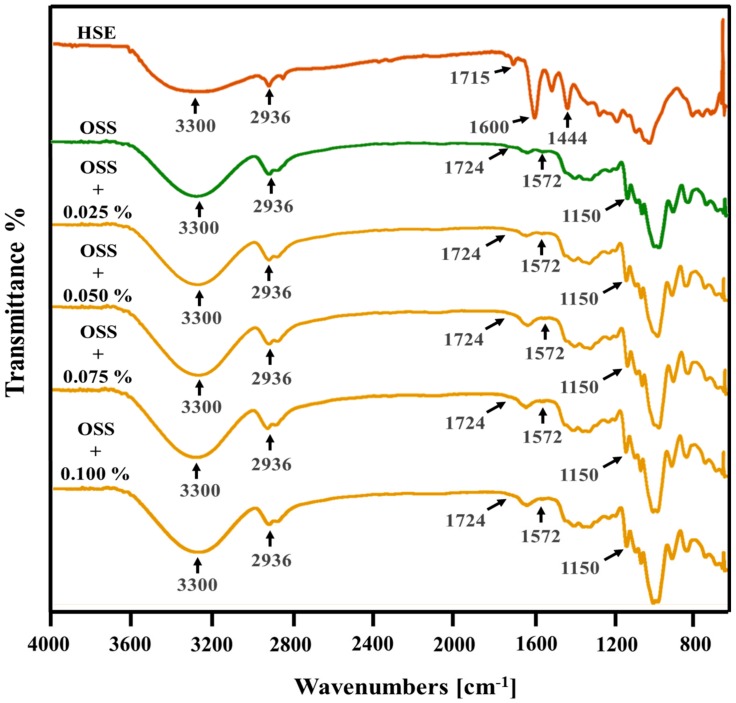
Fourier transform infrared (FTIR) spectra of hazelnut skin extract and octenyl succinate starch films with hazelnut skin extract. HSE: Hazelnut skin extract, OSS: octenyl succinate starch.

Surface and cross-section micrographs of OSS films by SEM are shown in Figure 4 and Figure 5**.** All films showed a similar surface, highlighting the absence of pores, holes, or cracks. This indicated that the extracts are dispersed throughout the structure of the starch and do not have any effect on the structural organization of the films [14,49]. However, some irregularities were observed with the presence of the extracts and the increase of concentration. This could indicate that maybe some solids or PCs were not completely dispersed in the starch network. The cross-section was similar in all films; however, irregularities were more evident in OSS films with PSE. These irregularities can influence the mechanical properties of films [43]. In this study, such irregularities could explain why OSS films with PSE presented differences in their mechanical properties compared with those OSS films with HSE (Table 6). In addition, the absence of layers in the cross-section images could indicate that extracts were dispersed homogeneously into the starch network. With respect to the surface topography, each of the films presented variable roughness (Figure 4 and Figure 5). The root mean square (RMS) in the OSS film was between the range of 72.03 to 166.10 nm. For OSS films with PSE, the range was from 72.03 to 484.44 nm, and for OSS films with HSE, the range was from 60.80 to 124.85 nm. The variation in the RMS indicated that some films have regions with greater roughness than others. This agreed with that observed in the surface micrographs (Figure 6 and Figure 7). The increase in RMS could also explain the increase in water resistance (hydrophobicity) of the films, specifically the contact angle and the water solubility (Table 3). This is because roughness allows for a greater amount of air to maintain contact with the film surface, which, consequently, reduces its interaction with water molecules [12,43]. 

### 3.7. Structural Analysis

Interactions between PC and OSS were identified by comparing the infrared spectrum of the extracts (PSE and HSE), OSS films, OSS films with PSE, and OSS films with HSE (Figure 6 and Figure 7). The PSE and HSE presented typical bands associated with PC, such as O-H stretching (3300 cm^−1^), C-H stretching for the aliphatic compounds or aldehyde groups, CH_2_ asymmetric stretching (2800–3000 cm^−1^ region), C=C stretching for aromatic rings (1600 cm^−1^)_,_ CH_2_ bending (1444 cm^−1^), and C-O and C-C bonds stretching (1000–1400 cm^−1^ region). These bands were similar to those reported by other authors [50,51]. In addition, both extracts showed bands close to 1510 cm^−1^ (C=C from aromatic rings) [14], and in the 900–700 cm^−1^ region, which could correspond to hydrogen atoms from aromatic rings [52]. The HSE presented more intense bands at the wavelength of 2800–3000 cm^−1^. There was a small band seen at 1715 cm^−1^, which could be related to the carbonyl [52] or C=O groups [45]. Also, there was a band close to 1271 cm^–1^, corresponding to C-O stretching [13]. 

This could be due to the differences in the PCs and their concentration in each extract. 

The OSS films presented typical bands from the starch molecule at 3330 cm^−1^ (OH groups), 2936 cm^−1^ (C-H stretching from the ring methane hydrogen atoms), 920–1160 cm^−1^ (C-O stretching) [53], and 1150 cm^−1^ corresponding to the glycosidic bond [52]. The bands at 1724 cm^−1^ and 1572 cm^−1^ could correspond to the C=O of the ester carbonyl groups and asymmetric stretching for the carboxylate group (RCOO-), respectively, as a result of the OSA modification [53]. The spectra of OSS films with PSE or HSE were similar, without presenting new peaks. This indicated that extracts were dispersed through the polymer matrix formed, as described by Eskandarinia, Rafienia, Navid and Agheb [43] and Feng, Yu, Zhu, Zhou, Liu, Yang, Zhou, Gao, Bao and Chen [14]. Despite this, some bands presented a slight increase in intensity in the presence of extracts. This could be a consequence of the intramolecular interaction between PC and starch through hydrogen bonds [14,45,46]. Overall, some PC were dispersed along the polymer matrix, while others were interacting directly with the starch structure mainly by hydrogen bonds.

## 4. Conclusions

The addition of pecan nutshell or hazelnut skin extracts partially improved the resistance to water by decreasing the solubility and increasing the contact angle (hydrophobicity), improving the UV-light blocking properties, and acting as plasticizing agents. It was observed that the phenolic compounds from extracts were complexed in the octenyl succinate starch matrix by hydrogen bonding, resulting in homogenous films. This indicated the potential use of octenyl succinate starch and nut by-products extracts as materials for the development of active films with applications in the food industry, such as packing material and edible films for foods that need to be stored under certain humidity conditions or UV protection. In addition, due to the antioxidant and antimicrobial properties of the phenolic compounds, these films should be evaluated using these properties against food spoilage, microorganisms, or pathogens of medical relevance to extend the application area of these films. 

## Figures and Tables

**Table 1:** Octenyl succinate starch films’ formulation.

Film Name	Film Forming Solution			Extract Solution	
	OSS (0.013) (g)	Glycerol (g)	Water (mL)	60% Ethanol Solution (mL)	PSE or HSE (mg per 100 mL)
OSS	4	2	90	10	-
OSS-PSE or HSE					
0.025%	4	2	90	10	25
0.050%	4	2	90	10	50
0.075%	4	2	90	10	75
0.100%	4	2	90	10	100

OSS: Octenyl succinate starch, PSE: pecan nutshell extract, HSE: hazelnut skin extract.

**Table 2:** Total phenols and flavonoids content in pecan nutshell and hazelnut skin extracts.

	PSE	HSE
Phenols Content (mg GAE/g)	656.46 ± 4.91 ^b^	693.00 ± 5.65 ^a^
Flavonoids Content (mg CE/ g)	235.35 ± 1.50 ^b^	427.77 ± 1.97 ^a^

Values represent a mean ± standard deviation, *n* = 3. Different letters in the same line indicate significant differences (*p* < 0.05). Tukey´s test. GAE: Gallic acid equivalents, CE: catechin equivalents, PSE: pecan nutshell extract, HSE: hazelnut skin extract.

**Table 3:** Thickness, moisture content, water-solubility, contact angle, and water vapor permeability of octenyl succinate starch films.

Film	Thickness (µm)	Moisture Content (%)	Water Solubility (%)	Contact Angle (°)	Water Vapor Permeability (g mm/ h m^2^ KPa)
OSS	87 ± 2 ^b^	29.54 ± 0.28 ^a^	20.34 ± 0.44 ^a^	49.25 ± 0.27 ^g^	2.46 ± 0.07 ^a^
PSE (%)					
0.025	90 ± 0 ^ab^	29.22 ± 0.54 ^a^	20.23 ± 0.30 ^a^	55.80 ± 1.79 ^f^	2.47 ± 0.05 ^a^
0.050	90 ± 1 ^ab^	29.29 ± 0.34 ^a^	19.39 ± 0.55 ^ab^	72.35 ± 0.34 ^e^	2.46 ± 0.06 ^a^
0.075	91 ± 1 ^a^	29.44 ± 0.78 ^a^	17.70 ± 0.56 ^c^	82.62 ± 0.88 ^c^	2.47 ± 0.08 ^a^
0.100	91 ± 1 ^a^	29.62 ± 0.83 ^a^	17.14 ± 0.21 ^c^	96.80 ± 0.21 ^a^	2.57 ± 0.08 ^a^
HSE (%)					
0.025	90 ± 1 ^ab^	29.83 ± 0.35 ^a^	20.72 ± 0.77 ^a^	70.38 ± 0.89 ^e^	2.59 ± 0.03 ^a^
0.050	90 ± 1 ^ab^	29.74 ± 0.40 ^a^	18.05 ± 0.72 ^bc^	77.45 ± 2.91 ^d^	2.61 ± 0.03 ^a^
0.075	91 ± 1 ^a^	30.03 ± 0.41 ^a^	17.78 ± 0.49 ^c^	83.96 ± 1.26 ^c^	2.59 ± 0.06 ^a^
0.100	91 ± 1 ^a^	29.90 ± 0.56 ^a^	17.08 ± 0.68 ^c^	92.54 ± 1.28 ^b^	2.62 ± 0.03 ^a^

Values represent a mean ± standard deviation, *n* = 3. Different letters in the same column indicate significant differences (*p* < 0.05). Tukey´s test. OSS: Octenyl succinate starch, PSE: pecan nutshell extract, HSE: hazelnut skin extract.

**Table 4:** Color parameters (L*, a*, b*, and *ΔE*) of octenyl succinate starch films.

Film	*L*	a*	b*	*ΔE*	*Color
OSS	95.09 ± 0.11 ^a^	−0.96 ± 0.02 ^f^	2.29 ± 0.04 ^g^	-	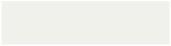
PSE %					
0.025	91.50 ± 0.35 ^b^	2.07 ± 0.29 ^e^	6.70 ± 0.52 ^f^	6.50 ± 0.68 ^e^	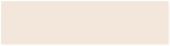
0.050	88.66 ± 0.05 ^c^	4.22 ± 0.10 ^d^	9.51 ± 0.38 ^de^	10.97 ± 0.30 ^d^	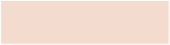
0.075	87.65 ± 0.52 ^c^	4.95 ± 0.39 ^d^	10.85 ± 0.37 ^d^	12.79 ± 0.72 ^d^	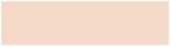
0.100	85.92 ± 0.52 ^d^	6.09 ± 0.60 ^c^	12.69 ± 0.74 ^c^	15.56 ± 1.01 ^c^	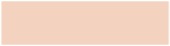
HSE %					
0.025	88.38 ± 0.34 ^c^	4.25 ± 0.29 ^d^	9.21 ± 0.49 ^e^	10.96 ± 0.66 ^d^	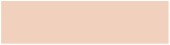
0.050	85.16 ± 0.61 ^d^	6.55 ± 0.48 ^c^	12.55 ± 0.69 ^c^	16.15 ± 1.00 ^c^	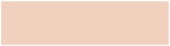
0.075	81.45 ± 0.41 ^e^	9.30 ± 0.31 ^b^	15.79 ± 0.51 ^b^	21.77 ± 0.65 ^b^	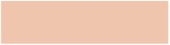
0.100	78.11 ± 0.71 ^f^	11.29 ± 0.55 ^a^	18.70 ± 0.60 ^a^	26.60 ± 0.96 ^a^	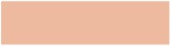

Values represent a mean ± standard deviation, *n* = 3. Different letters in the same column indicate significant differences (*p* < 0.05). Tukey´s test. OSS: Octenyl succinate starch, PSE: pecan nutshell extract, HSE: hazelnut skin extract, *ΔE*: color difference. *Color was obtained using the following website: https://www.nixsensor.com/free-color-converter/.

**Table 5:** UV-light and visible-light transparency of octenyl succinate starch films.

Film	T_280 nm_ (%)	T_600 nm_ (%)
OSS	71.24 ± 0.26 ^a^	86.17 ± 0.30 ^a^
PSE %		
0.025	22.76 ± 0.16 ^b^	84.32 ± 0.32 ^b^
0.050	11.08 ± 0.20 ^d^	83.81 ± 0.14 ^b^
0.075	3.07 ± 0.14 ^f^	82.75 ± 0.12 ^c^
0.100	1.58 ± 0.11 ^g^	80.26 ± 0.21 ^d^
HSE %		
0.025	14.39 ± 0.07 ^c^	84.36 ± 0.10 ^b^
0.050	3.56 ± 0.25 ^e^	83.82 ± 0.23 ^b^
0.075	0.61 ± 0.01 ^h^	82.32 ± 0.34 ^c^
0.100	0.22 ± 0.01 ^h^	80.22 ± 0.40 ^d^

Values represent a mean ± standard deviation, *n* = 3. Different letters in the same column indicate significant differences (*p* < 0.05). Tukey´s test. OSS: Octenyl succinate starch, PSE: pecan nutshell extract, HSE: hazelnut skin extract.

**Table 6:** Mechanical properties of octenyl succinate starch films.

Film	Puncture Resistance (N)	Tear Resistance (N)	Tensile Strength (MPa)	Elongation at Break (%)	Young´s Modulus (MPa)
OSS	4.48 ± 0.40 ^a^	1.36 ± 0.04 ^ab^	9.60 ± 1.38 ^ab^	32.41 ± 4.24 ^a^	239.55 ± 15.39 ^a^
PSE %					
0.025	3.62 ± 0.21 ^b^	1.12 ± 0.15 ^ab^	7.94 ± 0.78 ^ab^	30.98 ± 3.12 ^a^	154.00 ± 7.23 ^c^
0.050	3.65 ± 0.14 ^b^	1.11 ± 0.03 ^ab^	7.87 ± 0.42 ^ab^	25.90 ± 6.20 ^a^	171.26 ± 21.00 ^c^
0.075	3.79 ± 0.17 ^ab^	1.14 ± 0.25 ^ab^	7.56 ± 1.16 ^b^	23.99 ± 1.17 ^a^	159.06 ± 17.61 ^c^
0.100	3.58 ± 0.16 ^b^	1.00 ± 0.15 ^b^	8.62 ± 1.89 ^ab^	27.28 ± 6.93 ^a^	176.38 ± 19.83 ^bc^
HSE %					
250	4.01 ± 0.29 ^ab^	1.29± 0.07 ^ab^	9.36 ± 0.99 ^ab^	29.88 ± 1.89 ^a^	215.00 ± 5.30 ^ab^
500	3.79 ± 0.38 ^ab^	1.42 ± 0.18 ^ab^	10.58 ± 0.52 ^a^	29.80 ± 3.15 ^a^	224.85 ± 19.30 ^a^
750	3.79 ± 0.07 ^ab^	1.33 ± 0.06 ^ab^	8.35 ± 0.95 ^ab^	31.65 ± 4.75 ^a^	161.44 ± 11.58 ^c^
1000	4.04± 0.33 ^ab^	1.54 ± 0.26 ^a^	7.91± 0.93 ^ab^	29.58 ± 4.91 ^a^	161.20 ± 13.43 ^c^

Values represent a mean ± standard deviation, *n* = 3. Different letters in the same column indicate significant differences (*p* < 0.05). Tukey´s test. OSS: Octenyl succinate starch, PSE: pecan nutshell extract, HSE: hazelnut skin extract, N: Newtons, MPa: Mega Pascals.
